# Oxidative Stress Regulates a Pivotal Metabolic Switch in Dimethylsulfoniopropionate Degradation by the Marine Bacterium Ruegeria pomeroyi

**DOI:** 10.1128/spectrum.03191-22

**Published:** 2022-10-27

**Authors:** Tao Wang, Qiuyuan Huang, Andrew S. Burns, Mary Ann Moran, William B. Whitman

**Affiliations:** a Department of Microbiology, University of Georgia, Georgia, USA; b Department of Marine Sciences, University of Georgia, Athens, Georgia, USA; University of Minnesota

**Keywords:** marine bacteria, dimethylsulfoniopropionate, DMSP, oxidative stress, *Ruegeria pomeroyi*

## Abstract

Dimethylsulfoniopropionate (DMSP) is an abundant organic compound in marine surface water and source of dimethyl sulfide (DMS), the largest natural sulfur source to the upper atmosphere. Marine bacteria either mineralize DMSP through the demethylation pathway or transform it to DMS through the cleavage pathway. Factors that regulate which pathway is utilized are not fully understood. In chemostat experiments, the marine Roseobacter Ruegeria pomeroyi DSS-3 was exposed to oxidative stress either during growth with H_2_O_2_ or by mutation of the gene encoding catalase. Oxidative stress reduced expression of the genes in the demethylation pathway and increased expression of those encoding the cleavage pathway. These results are contrary to the sulfur demand hypothesis, which theorizes that DMSP metabolism is driven by sulfur requirements of bacterial cells. Instead, we find strong evidence consistent with oxidative stress control over the switch in DMSP metabolism from demethylation to DMS production in an ecologically relevant marine bacterium.

**IMPORTANCE** Dimethylsulfoniopropionate (DMSP) is the most abundant low-molecular-weight organic compound in marine surface water and source of dimethyl sulfide (DMS), a climatically active gas that connects the marine and terrestrial sulfur cycles. Marine bacteria are the major DMSP consumers, either generating DMS or consuming DMSP as a source of reduced carbon and sulfur. However, the factors regulating the DMSP catabolism in bacteria are not well understood. Marine bacteria are also exposed to oxidative stress. RNA sequencing (RNA-seq) experiments showed that oxidative stress induced in the laboratory reduced expression of the genes encoding the consumption of DMSP via the demethylation pathway and increased the expression of genes encoding DMS production via the cleavage pathway in the marine bacterium Ruegeria pomeroyi. These results support a model where DMS production in the ocean is regulated in part by oxidative stress.

## INTRODUCTION

Dimethylsulfoniopropionate (DMSP) is a ubiquitous low-molecular-weight organic compound in marine surface water produced mainly by marine algae, plants, corals, and bacteria (1, 2). DMSP is an osmolyte in many of these organisms and is often found in very high intracellular concentrations. In addition, it may act as an antioxidant, predator deterrent, and cryoprotectant (3–5). DMSP is also an important precursor of the volatile compound dimethyl sulfide (DMS) (6). The emission of DMS is the largest natural sulfur source to the upper atmosphere, where it participates in the formation of cloud condensation nuclei and is hypothesized to connect biotic activities and the global climate (7, 8).

The transformation of DMSP to DMS is accomplished through the cleavage pathway by algae, phytoplankton, and bacteria (9–11). So far, nine enzymes are known to possess this activity. They are from various enzyme families, have different catalytic mechanisms, and form different products, such as acrylate, acryloyl-CoA, or 3-hydroxypropionate-CoA (12–20). Bacteria have at least two additional DMSP metabolic pathways (21, 22). In the demethylation pathway, DMSP is processed by four enzymes, yielding methanethiol (MeSH) as the final product. MeSH can be directly captured for methionine biosynthesis or degraded to hydrogen sulfide for sulfur assimilation by many marine bacteria, and even at a low concentration of 0.3 μM, DMSP is still the preferred sulfur source (23, 24), which may explain why most DMSP is processed through the demethylation pathway (25). In the oxidation pathway, DMSP is first metabolized to dimethylsulfoxonium propionate (DMSOP) by both marine algae and bacteria. Bacteria can then degrade DMSOP to dimethyl sulfoxide (DMSO) and acrylate (22). However, the enzymes involved in this pathway are currently not known.

The enzyme catalyzing MeSH oxidation is MtoX (23). One by-product of this reaction is hydrogen peroxide (H_2_O_2_), a reactive oxygen species (ROS). The accumulation of ROS can potentially lead to oxidative stress, causing damage of cellular components, including lipids, proteins, and DNA. Compared to other ROS, H_2_O_2_ is not very reactive and is relatively stable. Because it is not charged, it can easily cross the cellular membrane (26). H_2_O_2_ is also common in marine surface water, where it is produced by solar radiation (27). The concentration of H_2_O_2_ in marine surface water is in the 0.02 to 0.80 μM range, although it might be higher near strong producers, such as phytoplankton (28, 29).

The antioxidant role of DMSP was first reported by Sunda et al. (30). They found that DMSP rapidly reacts with hydroxyl radicals and serves as a cellular scavenger. Moreover, the DMSP cleavage products acrylate and DMS as well as the DMS oxidation products dimethyl sulfoxide (DMSO) and methanesulfonic acid (MSNA) are also strong ROS scavengers. Thus, these molecules may form an effective antioxidant system, a role confirmed in algae, corals, and plants (31–35). However, there are few studies involving this process in bacteria, the major DMSP consumers (36–38).

**FIG 1:**
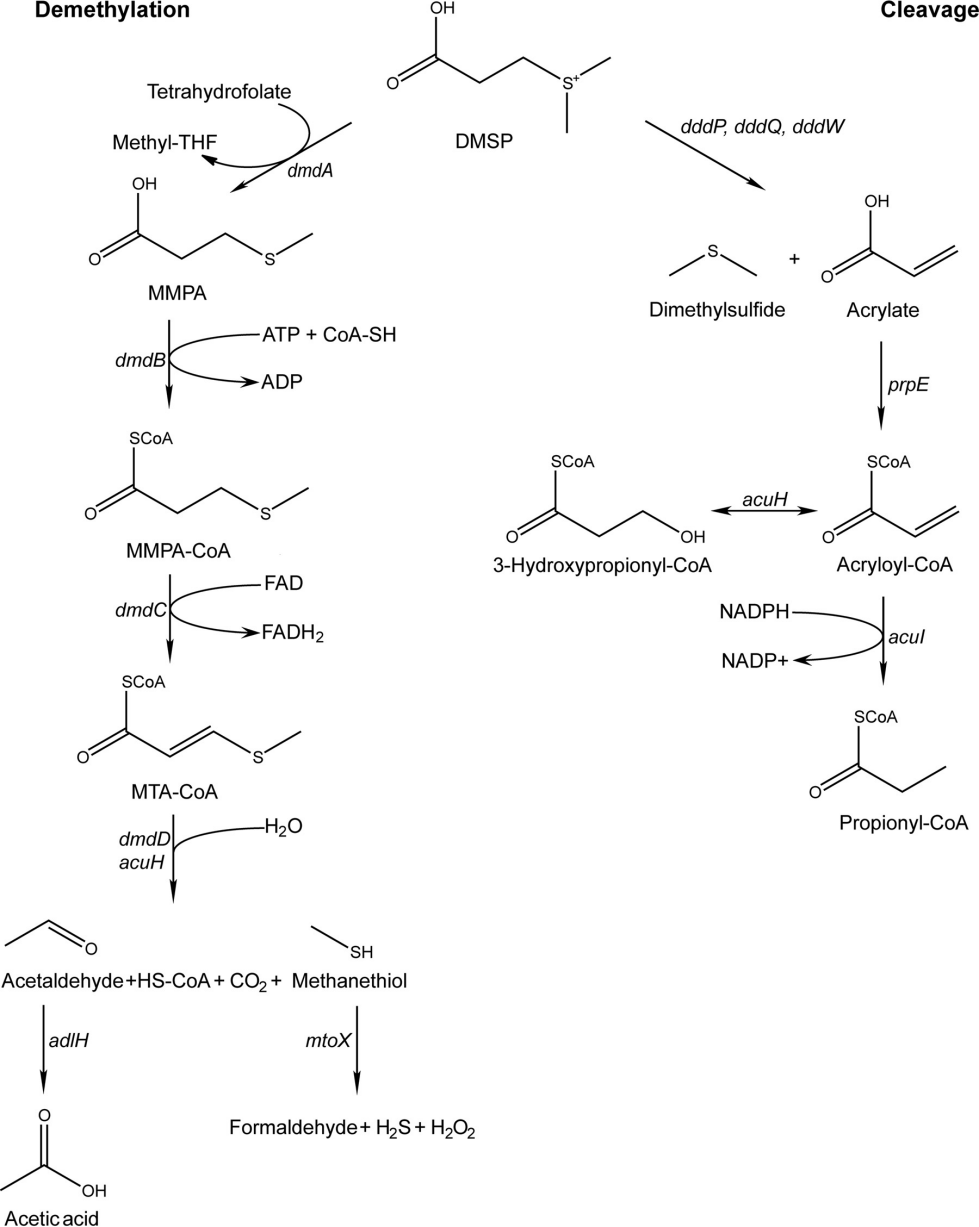
DMSP metabolic pathways in *R. pomeroyi* DSS-3. Genes: *dmdA* (SPO_RS09710), *dmdB* (SPO_RS10375, SPO_RS03420), *dmdC* (SPO_RS19300, SPO_RS01515, SPO_RS14785), *dmdD* (SPO_RS19305), *acuH*(SPO_RS00755), *adlH* (SPO_RS00490), *mtoX* (SPO_RS21180), *dddP* (SPO_RS11655), *dddQ* (SPO_RS22175), *dddW* (SPO_RS02290), *prpE* SPO_RS14880), and *acuI* (SPO_RS09715).

Bacterial metabolism of dissolved DMSP plays a major role in DMS evolution, with high levels of degradation by the demethylation pathway preventing DMS formation by the cleavage pathway, known as the “bacterial switch” hypothesis (39). However, the factors controlling the switch are poorly understood. Possibilities that have been identified include bacterial sulfur demand (6, 39, 40) and requirements for osmolytes (6, 41). In addition, oxidative stress has been proposed to play a role. ROS are common in marine environments, being generated by algae during photosynthesis as well as abiotically by photoreactions with dissolved organic matter. A role for ROS could explain the positive effect of UV light stress on DMS production (42, 43) and the correlation between expression of the genes encoding the demethylation pathway and catalase in Ruegeria pomeroyi and field populations of the Roseobacter HTCC2255 (36). To directly examine the role of oxidative stress in the bacterial switch, the relative expression of the genes encoding the demethylation and cleavage pathways was examined in chemostat cultures of *R. pomeroyi*. *R. pomeroyi* DSS-3 is a member of the *Rhodobacteraceae* and one of the most studied model organisms for DMSP catabolism. It possesses high activity for both cleavage and demethylation pathways (Fig. 1) (24, 40, 44). *R. pomeroyi* has three DMSP lyases, DddW, DddP, and DddQ (14, 16, 17). Among these three lyases, DddW is the one most upregulated by the presence of DMSP (45). In these experiments, oxidative stress was controlled by exposure to H_2_O_2_ in the wild type and a mutant with the gene encoding catalase (Δ*katG*) deleted, and the effects on transcription of the genes for DMSP metabolism were evaluated.

## RESULTS AND DISCUSSION

### Establishment of chemostat conditions.

One goal of the planned studies was to examine the relationship between oxidative stress and DMSP metabolism under conditions comparable to what might be found in nature, i.e., slow growth and low DMSP concentrations. For this reason, conditions were sought where the response to nonlethal concentrations of H_2_O_2_ could be determined during chemostat growth in both wild-type and a Δ*katG* mutant. In preliminary experiments in the absence of DMSP, 150 μM H_2_O_2_ rapidly killed chemostat cultures of the Δ*katG* mutant, but the wild-type tolerated at least 3 mM H_2_O_2_. At 100 μM and 1 mM H_2_O_2_, chemostat cultures of the mutant and wild type, respectively, were able to achieve steady-state growth, and these conditions were chosen for further study.

*R. pomeroyi* DSS-3 was previously cultivated in chemostats with a seawater-based minimal medium with 68 μM Fe(III)EDTA, an iron source required for growth (24). However, Fe(III)EDTA and other metal ions catalyze the degradation of H_2_O_2_ (46). To improve the stability of H_2_O_2_ throughout these experiments, a second reservoir was added to the chemostat system so that the H_2_O_2_ solution could be stored separately from the medium containing Fe(III)EDTA (see Fig. S1 in the supplemental material). In addition, to lower the rate of abiotic H_2_O_2_ decomposition in the chemostat, the Fe(III)EDTA concentration was lowered to 5 μM Fe(III)EDTA, which supported good growth and lower H_2_O_2_ decomposition rates.

Abiotic chemostat controls were performed to test the stability of DMSP and H_2_O_2_ under these conditions. In the abiotic chemostat, about 40 to 50% of the H_2_O_2_ added was consumed during the incubation, presumably due to a reaction with the remaining Fe(III)EDTA and other metal ions (Table S2). When DMSP was added, neither DMS nor MeSH was detected in the headspace, but the concentration of H_2_O_2_ in the outflow increased by about 10%. Likewise, the DMSP concentration declined by 7 to 8% regardless of the concentration of H_2_O_2_. The decomposition of H_2_O_2_ by Fe(III)EDTA is inhibited by hydroxyl radical scavengers (46). Because DMSP is a hydroxyl radical scavenger, its presence may have lessened abiotic H_2_O_2_ degradation.

### Chemostat growth and enzyme activities.

The medium for chemostat cultures contained 2 mM glucose with or without 0.20 mM DMSP. Under these conditions, *R. pomeroyi* consumed ≥99% of both substrates (Table 1) (24). The addition of DMSP resulted in a small increase in growth yield for wild-type but not Δ*katG* cultures. Upon addition of 1 mM H_2_O_2_ to the chemostat medium, the concentration of H_2_O_2_ remaining in the culture was <1 μM or not very different from 0.02 to 0.80 μM, the concentrations reported in surface seawater (28). For the Δ*katG* mutant, H_2_O_2_ consumption was severely impaired. Even upon addition of 110 μM H_2_O_2_ to the medium, 6 μM remained in the culture (Table 1). Similarly, upon the addition of 200 μM DMSP to the medium, most of it was consumed, and the amount remaining in the culture was near 2 μM (Table 1). While the DMSP concentration in open seawater is much lower, in the nM range, the concentration can reach μM levels, especially around phytoplankton cells (47, 48).

**TABLE 1:** DMSP consumption and metabolic data of *R. pomeroyi* strains during chemostat growth on glucose or glucose and DMSP before and during H_2_O_2_ additions^*a*^

Parameter	Unit	Wild-type		Δ*katG*	
		Glucose	Glucose + DMSP	Glucose	Glucose + DMSP
Inflow glucose	nmol min^−1^	200	200	200	200
Inflow DMSP	nmol min^−1^	0	20	0	20
Before adding H_2_O_2_^*b*^					
OD_600_	NA	0.30 ± 0.00	0.36 ± 0.01	0.33 ± 0.01	0.33 ± 0.01
Cell dry wt^*c*^	μg/mL	110 ± 0	133 ± 2	121 ± 2	122 ± 3
MeSH produced	nmol min^−1^	NA	0.04 ± 0.01	NA	0.06 ± 0.01
DMS produced	nmol min^−1^	NA	0.45 ± 0.02	NA	0.45 ± 0.08
Outflow DMSP	nmol min^−1^	NA	0.20 ± 0.01	NA	0.18 ± 0.01
Catalase activity^*d*^	μmol min^−1 ^mg^−1^	11.4 ± 0.5	6.9 ± 0.2	4.4 ± 0.3	1.8 ± 0.4
During H_2_O_2_ addition^*e*^					
Inflow H_2_O_2_^*f*^	μM	975	1045	110	107
OD_600_	NA	0.31 ± 0.01	0.30 ± 0.00	0.35 ± 0.01	0.34 ± 0.01
Cell dry wt	μg/mL	112 ± 2	112 ± 1	129 ± 3	126 ± 2
Outflow H_2_O_2_	μM	<1	<1	5.8 ± 1.3	<1
MeSH produced	nmol min^−1^	NA	0.06 ± 0.01	NA	0.05 ± 0.01
DMS produced	nmol min^−1^	NA	0.50 ± 0.05	NA	0.38 ± 0.06
Outflow DMSP	nmol min^−1^	NA	0.19 ± 0.02	NA	0.24 ± 0.04
Catalase activity	μmol min^−1 ^mg^−1^	24.3 ± 0.4	24.0 ± 0.5	1.8 ± 0.8	3.0 ± 0.4

a The 95% confidence intervals are based on three measurements unless indicated otherwise. NA, not applicable; OD_600_, optical density at 600 nm.

b The data reported are the means (*n* = 6) of results for the last 2 days of steady state before the addition of H_2_O_2_ except for the OD_600_, where *n* = 4. All cultures had a background H_2_O_2_ concentration of less than 1 μM.

c Cell dry weights were calculated from the absorbance at 660 nm using the following equation: dry weight = 364.74A_660_ + 6.7A_660_ × A_660_.

d For catalase-specific activity, 55% of the cell dry weight was assumed to be protein. Catalase activity was measured on whole cultures collected from the chemostat outflow, and the average of three determinations are reported. For the wild type, the catalase activity was significantly different before and after the treatment of H_2_O_2_ (*P* < 0.0001) and with and without DMSP (*P* < 0.05).

e After adding H_2_O_2_, all the parameters were measured three times daily. The data reported are the means (*n* = 9) of results for day 3 to day 5 after adding H_2_O_2_, except for OD_660_, where *n* = 6.

f The inflow H_2_O_2_ is calculated from the concentration measured in the reservoir.

The addition of H_2_O_2_ had little effect on growth yields and the production of MeSH and DMS. For instance, the differences upon H_2_O_2_ addition in culture optical density, MeSH and DMS production, and DMSP remaining observed in Table 1 were not significant according to *t* tests. Similarly, the differences between the wild-type and the Δ*katG* mutant were not significant. In contrast, H_2_O_2_ addition had a dramatic effect on the wild-type catalase-specific activity, which rapidly increased nearly 2-fold within the first day of exposure (Table S3). After 3 days, the catalase activity reached the maximum level and remained stable (Table 1). This result was consistent with a major role for catalase in the oxidative stress response. When DMSP was present in the absence of H_2_O_2_, the catalase-specific activity was reduced by about 40% (Table 1). Because DMSP inhibited the abiotic degradation of H_2_O_2_ in this medium, this result suggested that DMSP was able to mitigate oxidation stress independent of its effect on H_2_O_2_ concentrations. Upon addition of H_2_O_2_, the catalase-specific activity reached the same high level with or without DMSP (Table 1). Presumably, the high levels of exogenous H_2_O_2_ saturated the protective effect of DMSP.

For the Δ*katG* mutant, the observed catalase-specific activity in the absence of DMSP was about one-third of the wild-type levels (Table 1). As *katG* was the only catalase gene in *R. pomeroyi*, the catalase activity of the Δ*katG* mutant presumably reflected the activity of endogenous peroxidases or other enzymes. The specific activity did not increase upon addition of H_2_O_2_, a further indication that this activity was not mediated by KatG (Table 1). This activity was not sufficient to consume all the H_2_O_2_ added to the medium, even though the amount of H_2_O_2_ was only one-tenth of that added to wild-type cultures. Remarkably, addition of DMSP to the Δ*katG* culture led to a decrease in the levels of H_2_O_2_ (Table 1). Since DMSP inhibited the abiotic consumption of H_2_O_2_, the drop in extracellular H_2_O_2_ implied that DMSP stimulated a cell-dependent consumption of H_2_O_2_.

### Transcriptional response to DMSP and oxidative stress.

**FIG 2:**
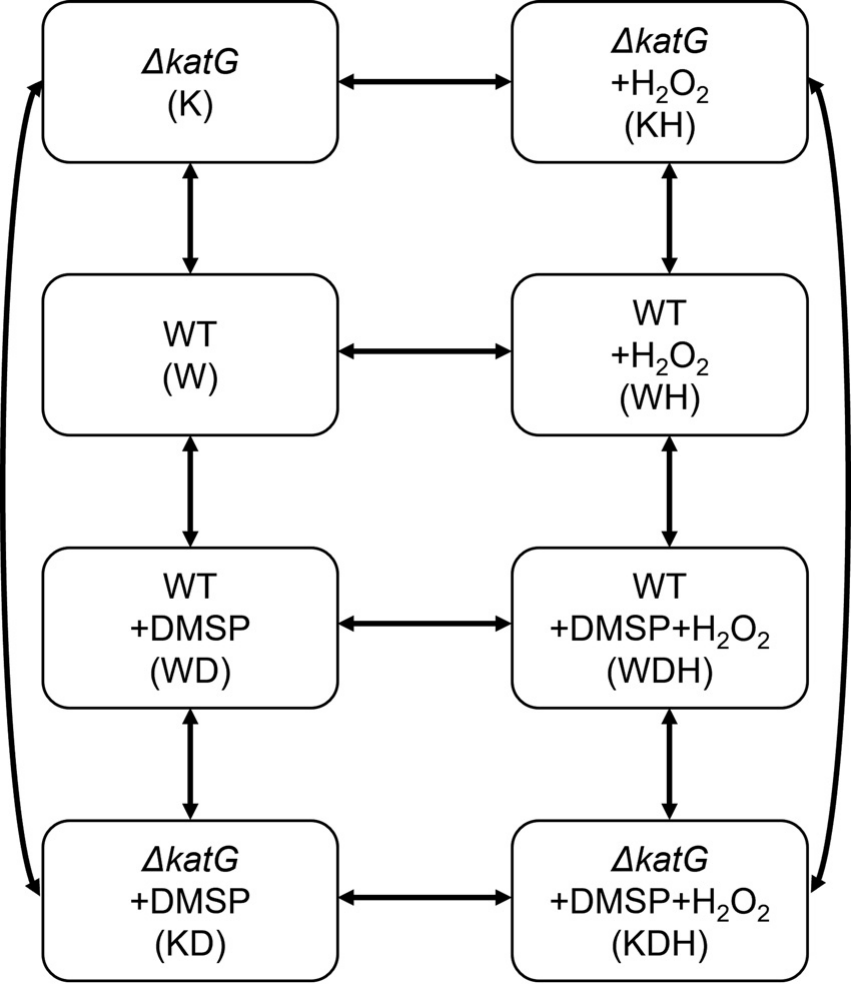
Experimental design for chemostat growth conditions. The arrows indicate the possible comparisons between selected conditions. Abbreviations for the growth conditions are indicated in parentheses. WT, wild-type *R. pomeroyi* DSS-3; Δ*katG*, the catalase deletion mutant.

To further elucidate the interaction between DMSP metabolism and oxidative stress, the transcriptional response was examined in 24 cDNA libraries representing 8 conditions, both wild-type and Δ*katG*, during growth on glucose, glucose plus DMSP, glucose plus H_2_O_2_, and glucose plus both DMSP and H_2_O_2_ (Fig. 2). Between 9.6 and 14.8 million (M) uniquely mapped clean reads were obtained for each library (Table S4). The reads of all 4,457 genes were counted using featureCounts and then processed using DESeq2. Replicates were also examined for differences due to batch effects, i.e., different chemostat runs, methods of RNA preparation, or sequencing runs. However, no evidence was found for systematic biases due to batch effects (supplemental material). The inclusion of one replicate (WD1) severely reduced the calculated number of differentially expressed genes (DEGs), and it was removed from subsequent analyses.

**FIG 3:**
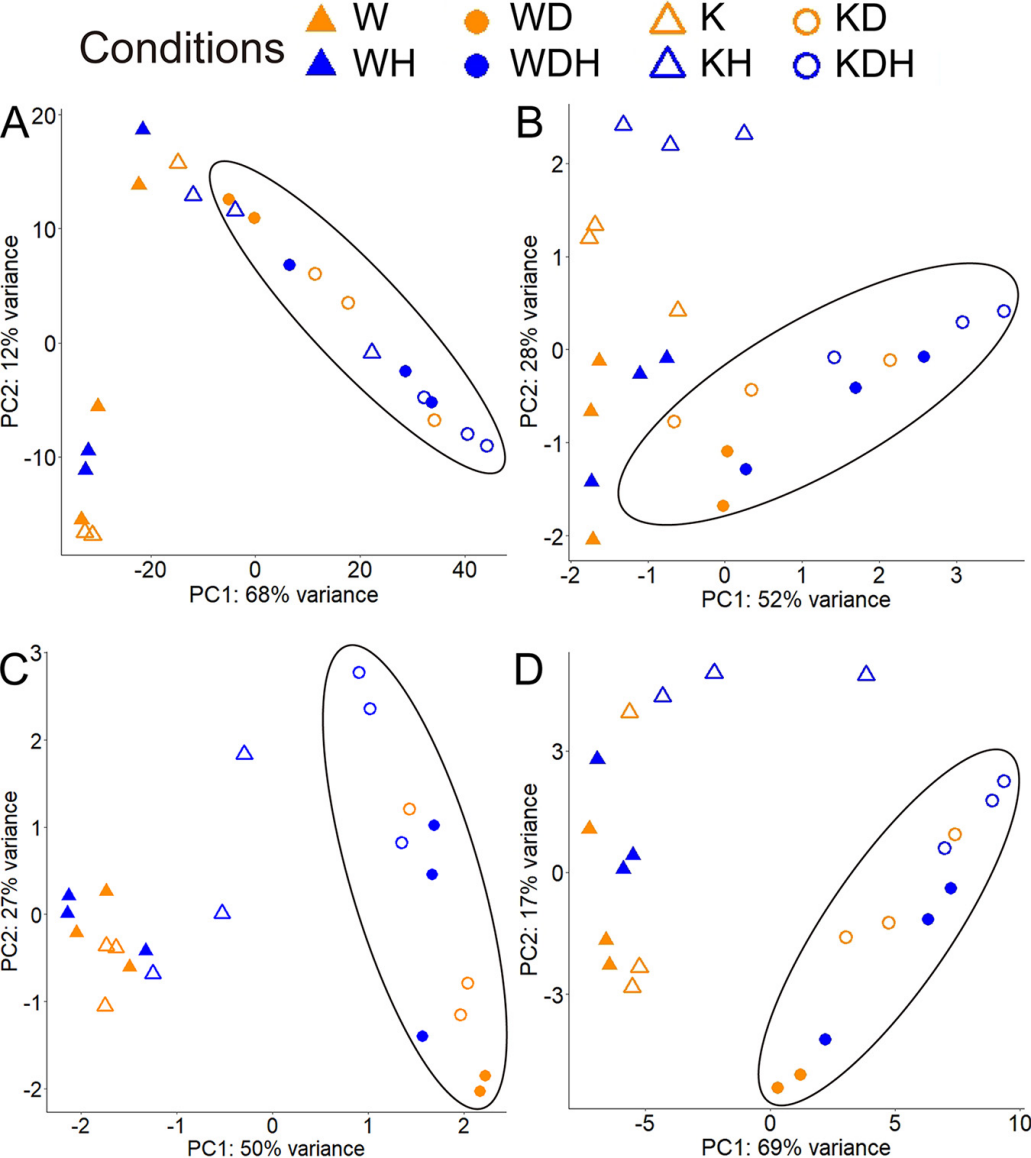
Effect of growth conditions on the patterns of gene expression. Principal-component analyses for RNA-seq results of each sample except for WD1. Samples containing DMSP are highlighted in ellipses. Abbreviations are defined in Fig. 2. (A) the entire genome; (B) oxidative stress genes listed in Table 2 (*katG* was not included because it is absent in the mutant); (C) DMSP metabolism genes listed in Table 2; (D) sulfur metabolism genes listed in Table 2.

In principal-component analysis (PCA) of the entire transcriptome, a strong signal was present for growth with DMSP (Fig. 3A). In addition, a smaller response of the Δ*katG* mutant to H_2_O_2_ was detected. To identify the physiological bases for these responses, the transcription of groups of functionally related genes was examined further.

### Identification of oxidative stress-responsive genes.

To examine the relationship between oxidative stress and DMSP metabolism, genes responsive to oxidative stress were first identified. While the oxidative stress response in *R. pomeroyi* has not been studied in detail, oxidative stress has been documented in some related proteobacteria, such as Rhodobacter sphaeroides (49, 50), and the OxyR regulon is a well-studied group of oxidative response genes whose function is conserved in proteobacteria (51). In addition, upregulation of genes encoding repair of DNA, proteins, and lipids is often associated with oxidative stress. Thus, the genome was searched for homologs to oxidative responsive genes from other proteobacteria, and 84 candidates were identified. Their response to H_2_O_2_ in both the wild type and the Δ*katG* mutant was then examined to determine their role in *R. pomeroyi* (Fig. S2). Many of these candidates had a complex or no response to the oxidative stressors used and were not suitable indicators. As an example, *R. pomeroyi* possessed four homologs to Escherichia coli *oxyR*, which encodes a transcription factor involved in the oxidative stress response in proteobacteria. However, only one of them, SPO_RS10675, had adequate abundance and was affected by the mutation or growth on H_2_O_2_. By these criteria, this gene and 17 others were chosen as indicators of oxidative stress in *R. pomeroyi* (Table 2).

**TABLE 2:** Fold change of expression of selected genes^*a*^

Gene	Locus tag^*b*^	Annotation	Fold change under growth condition:						
			WH	WD	WDH	K	KH	KD	KDH
Oxidative stress genes									
*katG*	20080	Catalase-peroxidase	2.120^*c*^	0.703	0.936	NA	NA	NA	NA
*soxR*	04980	Redox-sensitive transcriptional activator	0.614	0.909	0.620	0.630	0.452	0.497	0.233^*d*^
*soxS*	04985	Regulatory protein	0.935	3.218^*d*^	2.699^*d*^	0.855	0.949	1.394	2.021^*d*^
*sodB*	11860	Fe-Mn family superoxide dismutase	0.992	0.567	0.227^*d*^	0.686	0.769	0.451^*c*^	0.154^*d*^
*oxyR*	10675	H_2_O_2_-inducible genes activator	1.029	0.588	1.013	2.596^*d*^	2.153^*d*^	0.862	1.212
*lexA*	10920	SOS response genes repressor	0.851	0.391^*c*^	0.297^*d*^	2.871^*c*^	1.791	0.330^*d*^	0.203^*d*^
GPx	18990	Glutathione peroxidase	1.072	1.655	1.786^*c*^	3.228^*d*^	19.504^*d*^	1.722^*c*^	2.167^*d*^
*recA*	10320	Recombinase	0.701	0.595	0.357^*d*^	2.320	2.113	0.436^*c*^	0.162^*d*^
*mutT*	00305	8-oxo-dGTP diphosphatase	1.202	1.225	1.087	1.544	1.528^*d*^	1.323	1.061
*ruvB*	15785	Holliday junction DNA helicase	1.054	1.085	1.567^*d*^	2.136^*d*^	1.873^*d*^	1.223	1.620^*d*^
*ruvA*	15790	Holliday junction DNA helicase	1.063	0.965	1.488^*d*^	2.147^*d*^	2.156^*d*^	1.223	1.697^*d*^
*ruvC*	15795	Crossover junction endodeoxyribonuclease	1.412	1.460	3.448^*d*^	2.637^*c*^	5.608^*d*^	2.808^*d*^	4.431^*d*^
*uvrA*	11250	Excinuclease ABC subunit A	0.922	1.218	0.839	1.823	1.814^*d*^	0.825	0.511^*d*^
*uvrB*	02750	Excinuclease ABC subunit B	1.033	1.324	1.110	1.280	1.430^*d*^	0.951	0.883
*uvrD*	05950	Excinuclease ABC subunit C	0.760	0.672	0.590^*d*^	1.362	1.093	0.528^*d*^	0.386^*d*^
*sufD*	10235	Fe-S cluster assembly protein	1.069	0.548	0.422^*d*^	1.554	1.494	0.446^*d*^	0.427^*d*^
*sufC*	10240	Fe-S cluster assembly ATPase	1.317	1.179	0.890	2.150^*d*^	3.068^*d*^	0.987	0.744
*sufB*	10265	Fe-S cluster assembly protein	0.950	1.033	0.836	2.093	3.431^*d*^	0.971	0.415^*d*^
Demethylation pathway genes									
*dmdA*	09710	DMSP demethylase	1.347	2.309^*d*^	2.100^*d*^	1.277	1.066	2.406^*d*^	1.847^*d*^
*dmdB1*	10375	MMPA-CoA ligase	1.027	1.070	0.712	1.154	0.876	0.726	0.346^*d*^
*dmdB2*	03420	MMPA-CoA ligase	1.147	1.195	1.277	1.583^*c*^	0.867	1.234	1.421^*d*^
*dmdC1*	19300	MMPA-CoA dehydrogenase	0.910	3.422^*d*^	2.849^*d*^	1.180	1.819	3.719^*d*^	2.084^*d*^
*dmdC2*	01515	MMPA-CoA dehydrogenase	1.487	2.605	1.601	3.223	1.038	1.418	0.475
*dmdC3*	14785	MMPA-CoA dehydrogenase	1.114	3.957^*d*^	2.449^*c*^	2.127	0.967	2.099	0.734
*dmdD*	19305	MTA-CoA hydratase	1.476	4.966^*d*^	2.217^*d*^	1.038	2.012	3.535^*d*^	1.720
Cleavage pathway genes									
*dddP*	11655	DMSP lyase	0.744	1.004	1.069	0.700	0.709^*c*^	1.006	1.081
*dddQ*	22175	DMSP lyase	1.098	1.079	1.586^*c*^	0.911	0.828	1.257	1.874^*d*^
*dddW*	02290	DMSP lyase	2.169	4.304^*d*^	14.979^*d*^	1.943	8.304^*d*^	12.113^*d*^	20.800^*d*^
*acuI*	09715	NADPH-dependent acryloyl-CoA reductase	1.114	1.693	1.356	1.010	1.248	1.474	1.420
Other DMSP pathway genes									
*acuH*	00755	Enoyl-CoA hydratase	1.021	0.991	0.539	0.747	1.063	0.692	0.261^*d*^
*mtoX*	21180	MeSH oxidase	1.004	39.371^*d*^	13.000^*d*^	1.091	1.021	15.110^*d*^	4.171^*d*^
*adlH*	00490	Aldehyde dehydrogenase	1.364	1.077	1.082	1.092	0.855	0.827	1.042
*prpE*	14880	Propionyl-CoA synthetase	1.974	1.101	0.736	1.546	0.861	0.843	0.543^*d*^
Sulfate reduction genes									
*cysJI*	13360	Sulfite reductase	1.538	0.029^*d*^	0.056^*d*^	1.132	2.018	0.046^*d*^	0.088^*d*^
*cysH*	13365	Phosophoadenylyl-sulfate reductase	1.290	0.030^*d*^	0.079^*d*^	0.987	1.227	0.054^*d*^	0.094^*d*^
SAT	04535	Bifunctional sulfate adenylyltransferase/adenylylsulfate kinase	2.084	0.535	0.506	1.516	2.424	0.603	0.442
Sulfur oxidation *sox* enzyme system									
*soxV*	04990	Sulfur oxidation V protein	1.310	5.607^*d*^	21.655^*d*^	1.046	7.365^*d*^	14.899^*d*^	31.372^*d*^
*soxW*	04995	Thioredoxin	0.662	0.849	0.585^*c*^	0.901	0.450^*d*^	0.483^*d*^	0.310^*d*^
*soxX*	05000	l-cysteine S-thiosulfotransferase	0.710	1.890^*c*^	1.347	1.055	0.369^*d*^	0.474^*d*^	0.398^*d*^
*soxY*	05005	Sulfur oxidation Y protein	0.550	1.068	0.666	0.770	0.262^*d*^	0.325^*d*^	0.326^*d*^
*soxZ*	05010	Sulfur oxidation Z protein	0.620	0.999	0.462^*c*^	0.704	0.216^*d*^	0.171^*d*^	0.073^*d*^
*soxA*	05015	l-cysteine S-thiosulfotransferase	0.645	1.088	0.651	0.828	0.303^*d*^	0.344^*d*^	0.284^*d*^
*soxB*	05020	S-sulfosulfanyl-l-cysteine sulfohydrolase	0.682	2.754^*d*^	2.302^*d*^	0.845	0.409^*d*^	1.029	1.387
*soxC*	05025	Sulfur oxidation molybdopterin C protein	0.695	10.367^*d*^	10.058^*d*^	0.960	1.536	3.637^*d*^	5.234^*d*^
*soxD*	05030	S-disulfanyl-l-cysteine oxidoreductase	0.759	5.763^*d*^	5.591^*d*^	0.926	1.104	2.239^*d*^	3.523^*d*^
*soxE*	05035	Diheme cytochrome *c*	0.984	1.516	1.186	0.705	0.447^*d*^	0.692	0.829
*soxF*	05040	Sulfide-cytochrome *c* reductase	0.500^*d*^	0.733	0.638^*c*^	0.598	0.352^*d*^	0.319^*d*^	0.364^*d*^
Quinone-reducing molybdenum sulfite dehydrogenase SoeABC									
*soeC*	17005	Sulfite dehydrogenase subunit C	1.456	1.805	6.018^*d*^	0.981	2.885^*d*^	4.516^*d*^	8.861^*d*^
*soeB*	17010	Sulfite dehydrogenase subunit B	0.856	1.208	1.664^*c*^	1.118	1.245	1.423	2.160^*d*^
*soeA*	17015	Sulfite dehydrogenase subunit A	1.067	1.205	3.568^*d*^	1.633	4.533^*d*^	3.150^*d*^	5.716^*d*^
Methionine and cysteine metabolism genes									
*metY*	07295	*O*-acetyl-l-homoserine sulfhydrylase	1.379	0.879	0.456^*d*^	0.834	0.706	0.578^*d*^	0.317^*d*^
*metZ*	06880	*O*-succinyl-l-homoserine sulfhydrylase	1.385	0.704	1.019	0.929	2.006	1.098	1.499
*megL*	21430	Methionine gamma-lyase	1.235	4.841^*c*^	38.284^*d*^	1.242	14.694^*d*^	27.219^*d*^	58.386^*d*^
MTR	09575	5-methyltetrahydrofolate-homocysteine methyltransferase	0.736	0.515^*c*^	0.336^*d*^	1.086	0.560^*d*^	0.368^*d*^	0.203^*d*^
*cysQ*	00195	3′(2′),5′-bisphosphate nucleotidase	1.228	1.330	0.947	0.746	1.265	1.236	0.628
*cysK*	11395	Cysteine synthase A	1.211	0.367^*d*^	0.193^*d*^	0.929	1.392	0.184^*d*^	0.104^*d*^
*cysE*	11400	Serine *O*-acetyltransferase	0.960	0.773	0.410^*d*^	0.947	0.704	0.426^*d*^	0.166^*d*^
Other gene									
*dddD*	08640	DMSP lyase DddD-like gene	0.911	1.105	2.480^*d*^	1.000	1.390	2.958^*d*^	4.846^*d*^

a Abbreviations for growth conditions are defined in Fig. 2. Each comparison was made to the wild type with no additions or W transcriptome. Conditions in parentheses are controls for each comparison. NA, could not be compared due to the gene deletion.

b All locus tags have the prefix SPO_RS.

c Adjusted *P* value, <0.1.

d Adjusted *P* value, <0.05.

**FIG 4:**
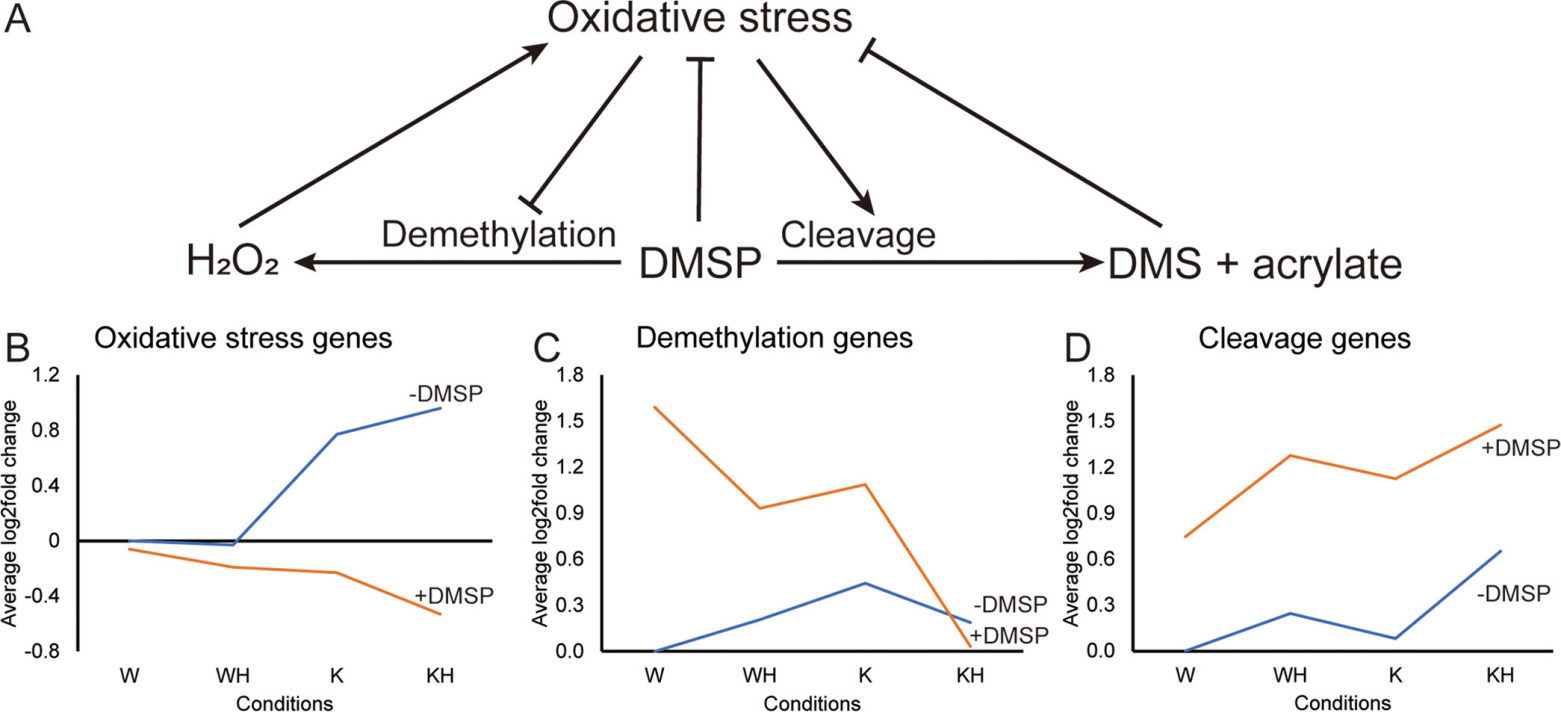
Regulation network of oxidative stress on DMSP metabolism based on changes in gene expression. The increasing expression of oxidative stress genes from W, WH, K, and KH when DMSP was not present indicated the increasing oxidative stress among the conditions. The log_2_ fold changes of selected genes were calculated compared to wild type with no additions (the W transcriptome). (A) Proposed regulation network: DMSP demethylation leads to H_2_O_2_ production and oxidative stress. Oxidative stress downregulates the demethylation pathway and upregulates the cleavage pathway, leading to the production of strong antioxidants that reduce oxidative stress. (B) Average log_2_ fold change compared to W of the oxidative stress genes listed in Table 2. (C) Average log_2_ fold change compared to W of the demethylation pathway genes listed in Table 2. (D) Average log_2_ fold change compared to W of the cleavage pathway genes listed in Table 2.

Expression of the oxidative stress-responsive genes was affected by growth in the presence of DMSP and H_2_O_2_ as well as the Δ*katG* mutation. In PCA, the pattern of transcription of the oxidative stress-responsive genes in cultures grown with DMSP was clearly distinguished from that of those grown without. Smaller effects were also seen for growth with H_2_O_2_ and between the wild type and the Δ*katG* mutant. The expression of *katG* in the wild type increased nearly 2-fold upon addition of H_2_O_2_, comparable to the increase in specific activity during growth with H_2_O_2_ (Table 2). The expression of seven of the indicator genes increased in the mutant compared to the wild type (Table 2, Fig. 4). These included the genes for DNA and Fe-S cluster repair in the *ruv* and *suf* operons, respectively. The low expression of these repair genes in the wild type even with H_2_O_2_ addition suggested that the wild-type strain was well protected against H_2_O_2_ exposure. Lastly, expression of Gpx, an important enzyme reducing hydroperoxides, including lipid hydroperoxide (52), and *ruvC*, a gene involved in DNA repair, further increased in the mutant upon addition of H_2_O_2_. These increases were evidence for the high susceptibility of the mutant to H_2_O_2_.

The addition of DMSP had strong effects on the expression of the oxidative stress-responsive genes, especially in the mutant (Table 2). During growth of the mutant with DMSP, there was a 2- to 8-fold reduction in the expression of many of the oxidative stress-responsive genes in both the presence and absence of H_2_O_2_. The results with the wild type were similar, although fewer genes were affected. Similarly, for the wild type the specific activity of catalase was greatly reduced during growth with DMSP (Table 1). These results indicated that DMSP or the intermediates or products of its metabolism were largely protective against oxidative stress.

### Interaction between oxidative stress and DMSP metabolism.

In the absence of DMSP, the DMSP metabolic genes are only transcribed at low levels (45). This low-level or basal expression in both the wild type and mutant was unaffected during growth with H_2_O_2_ (Fig. 3C). Upon growth with DMSP, expression of genes encoding both the demethylation and cleavage pathways increased (Table 2, Fig. 4). The largest increases were in the expression of *mtoX* and the demethylation genes, *dmdD*, *dmdC1*, and *dmdC3*. Among the cleavage genes, the largest increases were in *dddW*. The addition of H_2_O_2_ lowered the expression of the genes encoding the demethylation pathway in both the mutant and wild type. In contrast, the expression of *dddW* of the cleavage pathway increased in the wild type (Table 2). Likewise, compared with the wild type, the mutant had significantly higher expression of *dddW* but lower expression of *mtoX*. In addition, the *dddD*-like gene SPO_RS08640 was also significantly downregulated. Thus, oxidative stress lowered the expression of the genes of the demethylation pathway and increased expression of the genes of the cleavage pathway (Fig. 4).

### Response of sulfur metabolism to DMSP and oxidative stress.

**FIG 5:**
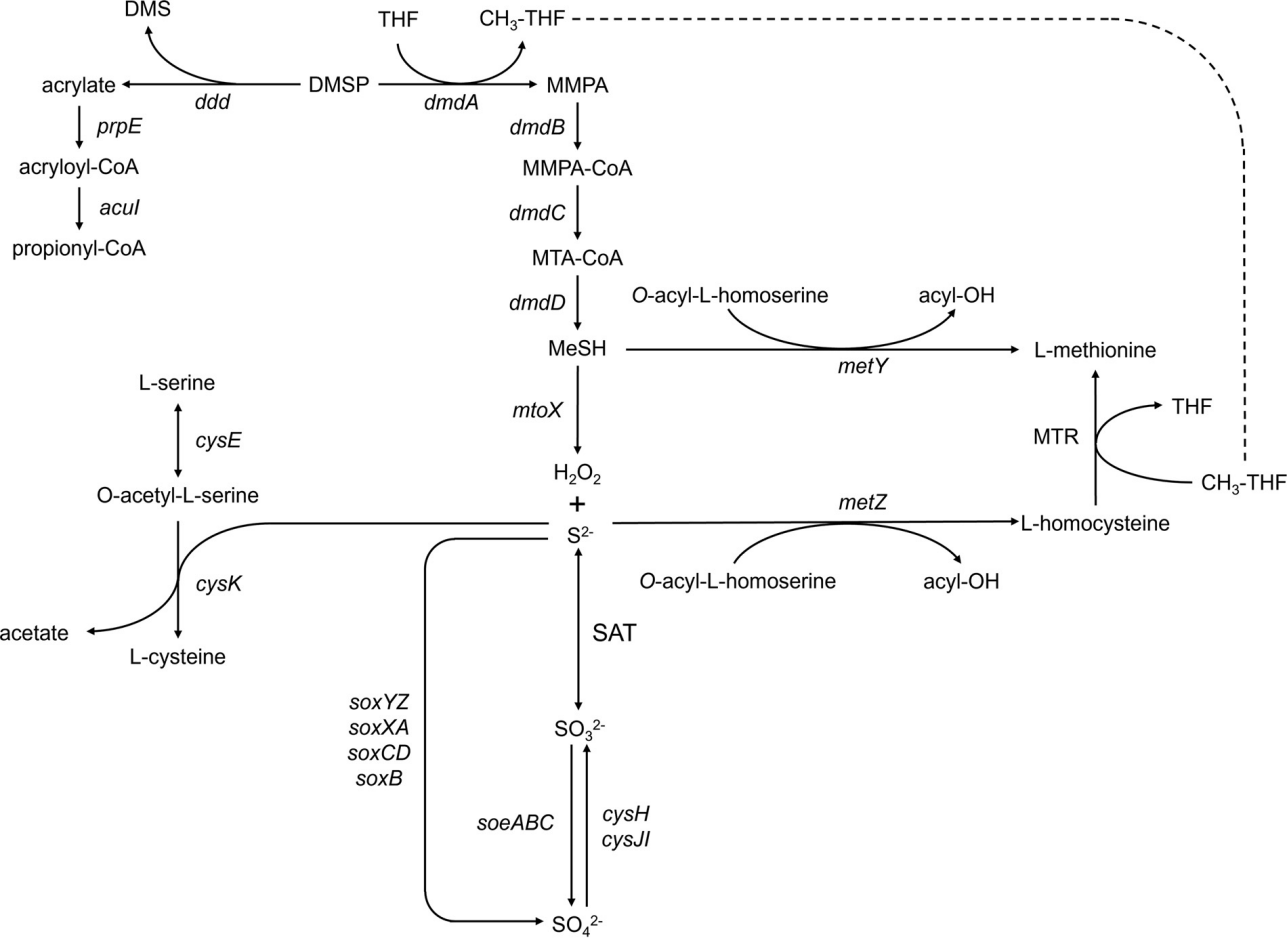
Overview of sulfur metabolism in *R. pomeroyi*. Sulfide can be used for biosynthesizing cysteine or methionine by CysK or MetZ and MTR, respectively. Sulfide can be produced either by reducing sulfate or oxidizing MeSH released during DMSP demethylation. MeSH can also be directly incorporated for methionine biosynthesis by MetY. For locus tags, see Tables 2.

DMSP is a preferred sulfur source for *R. pomeroyi* (24), and growth with DMSP dramatically changed the pattern of expression of the sulfur metabolism genes (Fig. 3D). DMSP sulfur is incorporated via the demethylation pathway, where both sulfide and MeSH are intermediates for biosynthesis of the sulfur-containing amino acids (Fig. 5). When grown only on glucose, sulfate was the only sulfur source available, and the expression of the genes involved in sulfate assimilation was high. Upon addition of DMSP, the expression of the sulfate assimilation genes was greatly reduced, as expected if DMSP was the primary sulfur source (Table 2). Expression of *metY*, which encodes an alternative route of biosynthesizing methionine from MeSH, also declined (Table 2). Similarly, expression of 5-methyltetrahydrofolate-homocysteine methyltransferase (MTR), cysteine synthase A (*cysK*), and serine *O*-acetyltransferase (*cysE*) declined during growth on DMSP (Table 2). Possibly, when intracellular MeSH and sulfide are abundant, lower levels of these enzymes are required to satisfy the requirement for biosynthesis of methionine and cysteine. Increases in oxidative stress either in the mutant or by the addition of H_2_O_2_ did not cause major changes in the expression of the genes encoding sulfate assimilation. This result suggests that under oxidative stress, DMSP would likely remain the primary sulfur source.

Previous studies have shown that during growth with DMSP as the sole carbon source, the expression of the *sox* genes for sulfur oxidation was upregulated (53). The *sox* genes of *R. pomeroyi* are mainly present in a single cluster as *soxVWXYZABCDEF*, encoding enzymes oxidizing various inorganic sulfur compounds (54, 55). When grown with DMSP, the expression of *soxC* and *soxD* increased significantly in both the wild type and mutant (Table 2). These genes encode the enzyme Sox(CD)_2_, which plays an important role in sulfur oxidation. Likewise, expression of *soxV*, a membrane protein involved in sulfur oxidation, also increased. These increases in expression would facilitate metabolism of sulfide during DMSP demethylation. However, the increases in their expression as well as that of many of the other *sox* genes was lower in the Δ*katG* mutant than the wild type (Table 2). This pattern was consistent with the decreased expression of the genes encoding the demethylation pathway in the mutant. Likewise, SoeCBA is a cytoplasmic sulfite dehydrogenase (56) whose expression increased during growth with DMSP in the mutant but did so in the wild type only when H_2_O_2_ was also present (Table 2 and data not shown). Presumably, the increased expression of *soeCBA* was a response to increased sulfite formed from sulfide under oxidative stress. Similarly, the expression of *megL* encoding methionine gamma-lyase greatly increased upon exposure to H_2_O_2_ in both the wild type and the mutant. This enzyme may play a role in metabolism of methionine sulfoxide, a common oxidation product of methionine, and may be protective of oxidative stress (57, 58).

### Conclusions.

In the mixed substrate chemostat used in these experiments, conditions were chosen to simulate those close to what might be found under some natural conditions; i.e., the DMSP concentration in the culture was maintained at a low value of about 2 μM, the growth rate was slow with a doubling time of 24 h, and the cell density remained constant. In addition, the rates of DMSP consumption and DMS and MeSH production were similar in both the wild type and mutant in the presence and absence of H_2_O_2_. Thus, in spite of the changes in the transcriptome, DMSP metabolism was not affected by the levels of oxidative stress induced by these manipulations. Many studies have reported a low correlation between the abundance of mRNAs and proteins, indicating the importance of posttranscription regulation, translational regulation, degradation, and other regulatory processes (59). Thus, changes in gene expression and enzyme levels will only affect metabolism if they change the levels of key, rate-limiting enzymes. Nevertheless, the transcriptome still provides insights into the factors sensed by the cells and used to control metabolism.

The demand for reduced sulfur has also been proposed to be a major regulatory strategy for DMSP metabolism in *R. pomeroyi*. However, under the conditions used here, the sulfur demand would account for only a small fraction of the DMSP consumed by the demethylation pathway. For instance, in the chemostat culture with 2 μM DMSP, the rate of DMSP consumption by wild-type cells was 19.8 nmol min^−1^, 0.45 nmol min^−1^ of which was consumed by the cleavage pathway and 19.3 nmol min^−1^ of which was consumed by the demethylation pathway. In contrast, the sulfur demand was about 3.4 nmol min^−1^ or about 18% of the activity of the demethylation pathway. Thus, the rate of demethylation far exceeded the sulfur demand at these concentrations. Similar results have been found at other DMSP concentrations in carbon-limited chemostats (Table 3). Even at lower extracellular DMSP concentrations of 0.3 μM, only 36% of the demethylation activity was needed to satisfy the sulfur demand, and at 61 μM only 1% of the demethylation activity was needed (24). Lastly, under both 0.3 and 2 μM DMSP, the rate of DMS production was nearly the same, implying that the cleavage activity was independent of the relative sulfur demand, and sulfur demand played no more than a minor role in the regulation of DMSP metabolism at these concentrations in *R. pomeroyi*.

**TABLE 3:** DMSP consumption by *R. pomeroyi* during chemostat growth on glucose plus DMSP^*a*^

Parameter	Unit	100 μM^*b*^	200 μM	5,000 μM^*b*^
Inflow DMSP	nmol min^−1^	10	20	500
DMSP concn remaining	μM	0.3	2.0	61
DMSP metabolized	nmol min^−1^	10.0	19.8	494
Demethylation activity	nmol min^−1^	9.6	19.3	400
Cleavage activity	nmol min^−1^	0.4	0.5	93.6

a Chemostats were carbon-limited and fed 200 nmol min^−1^ (or 2 mM) glucose plus the indicated amounts of DMSP. Dilution rates were 1 Da^−1^ in minimal medium as described in Table 1 and Materials and Methods.

b Calculated from the data in Table 1 of Wirth et al. (24). The chemostats were run under the same conditions as this study but with 68 μM Fe(III)EDTA.

There is a complex interaction between DMSP metabolism and oxidative stress in *R. pomeroyi*. In response to DMSP, expression of the indicators of oxidative stress are downregulated, suggesting that DMSP is largely protective. In response to oxidative stress, the DMSP transcriptome shifts from the demethylation to the cleavage pathway. Like many marine roseobacters, *R. pomeroyi* is adapted to live associated with marine phytoplankton (60). Phytoplankton, including diatoms, dinoflagellates, and coccolithophores, produce both DMSP and ROS, such as superoxide and H_2_O_2_ (11, 61, 62). Thus, it is likely that when *R. pomeroyi* grows in the phycosphere, it is exposed to higher concentrations of both DMSP and H_2_O_2_ than are found in open seawater (63), and cells must balance the response to both compounds. In this context, the cleavage pathway produces the strong ROS scavengers acrylate and DMS and, unlike the demethylation pathway, does not produce the oxidant H_2_O_2_. For these reasons, it would be favored during oxidative stress.

Under the experimental conditions chosen, both the expression of the *katG* gene and the catalase activity declined during growth with DMSP. In contrast, Varaljay et al. (36) observed an increase in *katG* expression when *R. pomeroyi* was exposed to DMSP. However, the concentration of DMSP was 80 μM, much higher than used here. Presumably, the higher levels of DMSP may have led to increased demethylation activity and higher production of intracellular H_2_O_2_. The levels of H_2_O_2_ and not DMSP may have then regulated *katG* expression, a conclusion consistent with increases in KatG activity observed here during growth with H_2_O_2_.

These results are consistent with the hypothesis that at the concentrations of DMSP investigated, oxidative stress is one of the factors controlling the bacterial switch between the demethylation and cleavage pathways in *R. pomeroyi* (Fig. 4). In this model, demethylation activity increases with increasing concentrations of DMSP, and with it, the intracellular concentration of H_2_O_2_ increases. The higher levels of H_2_O_2_ lead to increases in oxidative stress, which then provides the signal for increased expression of the cleavage pathway, which not only reduces further H_2_O_2_ production but also produces more efficient scavengers for ROS, such as acrylate and DMS. Evidence to support this model include the following. (i) Expression of the cleavage pathway only becomes significant in cultures grown at very high concentrations of DMSP, well beyond the amount needed to satisfy bacterial sulfur demand. This result seems to preclude the sulfur demand hypothesis. (ii) Moreover, it is generally consistent with the observations of Gao et al. (45), who reported that the relative expression of a cleavage pathway gene increases at high DMSP concentrations. (iii) Growth with DMSP reduces the expression of oxidative stress-responsive genes, suggesting that DMSP protects against oxidative stress. (iv) Oxidative stress induced by the addition of H_2_O_2_ or the *katG* mutation reduces the expression of the demethylation genes and increases the expression of the cleavage genes.

Other regulatory schemata are also likely to play a role. For instance, acrylate, the product of the cleavage pathway, is both toxic and a strong scavenger of hydroxyl radicals. Its toxicity seems to be the basis of a complex interaction between the two DMSP catabolic pathways. *dmdA*, the first gene of the demethylation pathway, and *acuI*, which encodes the reduction of acryloyl-CoA to propionyl-CoA, share an operon that is coregulated by both DMSP and acrylate (64, 65). Loss of a functional *acuI* abolishes *R. pomeroyi*’s ability to grow on DMSP or acrylate as the sole carbon source. This indicates that acrylate or acryloyl-CoA accumulated via the cleavage pathway is highly toxic and needs to be removed, even though this pathway only processes a small portion of the DMSP (24, 65). Moreover, addition of even small amounts of acrylate to cultures leads to higher production of MeSH and DMSP demethylation activity (37). Thus, this regulatory network may serve to limit the cleavage pathway and DMS production.

The regulation of bacterial DMSP degradation in ocean surface waters has been a focus of oceanographic and atmospheric science research for several decades (7, 39). Although the negative feedback between the biogenic DMS production in the upper-ocean ecosystem and climate, proposed as the CLAW hypothesis, has not been supported by recent studies (66), the role of DMS in producing cloud condensation nuclei is well recognized. Field and modeling data indicate that up to 50% of DMSP produced in some systems is routed to climate-active DMS via the cleavage pathway (6). However, DMS production represents a loss of reduced carbon and sulfur to the bacterium, and the rationale for its production by marine bacteria has not been determined. Here, we show that oxidative stress in marine bacteria can be a determining factor for the metabolic fate of DMSP. Thus, the cleavage pathway may have evolved as a means of reducing oxidative stress. It has been reported that the production of DMS from DMSP has a strong seasonality in marine environments (67, 68). One of the explanations is the high UV radiation during summer inhibits the growth of marine bacteria so that both the demand for sulfur and the consumption of DMS decrease (42). Our results show that under the oxidative stress, potentially caused by UV radiation, bacteria also tend to increase the production of DMS. This could be another explanation for the seasonality of DMS production and rationale for the widespread distribution of the cleavage pathway. Overall, our study connects the activities of marine bacteria to the complex dynamic of the global climate.

## MATERIALS AND METHODS

### Construction of catalase deletion stain (Δ*katG*).

A deletion mutant of *katG* was constructed by homologous recombination as described previously (69). Briefly, 1-kb regions up- and downstream of the *katG* gene in the *R. pomeroyi* genome and *tetAR* cassette from pRK415 were cloned into pCR2.1 using sequence- and ligation-independent cloning (SLIC) (70) and electroporated into competent *R. pomeroyi* cells using a BTX Electro cell manipulator 630 under the following conditions: 1.8 kV, 24 μF, 200 Ω. Recombinant clones were selected on tetracycline-amended (20 μg/mL) 1/2YTSS medium (DSMZ medium 974), and the *katG* deletion was verified via PCR and sequencing. The primers used are listed in Table S1.

### Chemostat cultivation.

*R. pomeroyi* DSS-3 was routinely cultivated at 30°C on a carbon-limited chemostat with modification, where two reservoirs were used to improve the stability of H_2_O_2_ (Fig. S1) (24, 71). Chemostat experiments were initiated with inoculation of a frozen glycerol stock culture of *R. pomeroyi* into 1/2YTSS broth (with tetracycline for the Δ*katG* strain) in a shaking incubator at 30°C. After 24 h, 1 mL of the starter culture was inoculated into the empty chemostat vessel, and the addition of fresh medium was started. The medium was supplemented with sufficient glucose or glucose plus DMSP for final concentrations of 2 mM glucose and 0.2 mM DMSP.

For cultures with DMSP, MeSH and DMS present in the headspace of the chemostats and DMSP remaining in the outflow were measured twice daily (24, 53). The DMS and MeSH concentrations in the aqueous phase were then calculated using the distribution coefficient for 10 ppm DMS (*K_i_* = 8.830) or MeSH (*K_i_* = 7.107) at 30°C in artificial seawater (72). Outflow DMSP was measured by mixing 1 mL of chemostat outflow with 1 mL of 4 M NaOH in a crimp-sealed vial (actual volume, 11.5 mL) to hydrolyze DMSP to DMS. Then, 1 mL of headspace gas was analyzed after 0.5 h of incubation at 30°C. The DMSP in the outflow was calculated based on the injected DMS and the distribution coefficient mentioned above.

To add H_2_O_2_ into the chemostat, 2 mM or 0.2 mM H_2_O_2_ was applied to the phosphate solution for wild-type or Δ*katG* strains, respectively, when the culture reached steady state. At the end of each experiment, contamination of the chemostat was checked by sequencing the PCR-amplified 16S rRNA genes (73). No contamination was found for all chemostats.

### RNA extraction and sequencing.

The chemostat outflow for RNA extraction was collected in prechilled 5% (wt/vol) phenol-ethanol solution on ice to stabilize mRNA. After 4 h, the collections were centrifuged at 5,000 × *g* for 20 min at 4°C. Cell pellets were either stored at −80°C or processed for RNA extraction immediately. For each condition, three samples were collected. Total RNA extraction and DNA digestion were performed using the ZymoBIOMICS RNA miniprep kit (Zymo Research) with elongated bead beating. The quality of purified total RNA was checked with agarose gel electrophoresis, Qubit RNA high-sensitivity (HS) assays (Invitrogen), and a NanoDrop instrument. Some samples were further purified with the RNA Clean & Concentrator kit (Zymo Research). The eligible samples were sent to Novogene for the subsequent steps. rRNA was removed using the Ribo-Zero kit. Strand-specific cDNA libraries were prepared and sequenced using an Illumina NovaSeq 6000 sequencing system with a read length of PE150.

### Bioinformatic analysis.

Raw data were trimmed with Trim Galore to remove adapters and reads with quality scores lower than 20 (74). Reads were mapped to the *R. pomeroyi* DSS-3 genome using Bowtie2 (version 2.4.2) and counted using featureCounts (version 2.0.1) (75, 76). Differential expression of genes between each condition was calculated using DESeq2 (version 3.12) in R (version 4.0.5) with *apeglm* type shrinkage of log_2_ fold changes (77, 78). For details, see the supplemental material.

### Data availability.

Data that support the findings of the present study have been deposited in the National Center for Biotechnology Information’s Sequence Read Archive under BioProject number PRJNA828625.
