# Supplementary material for: Oxidative Stress Regulates a Pivotal Metabolic Switch in Dimethylsulfoniopropionate Degradation by the Marine Bacterium Ruegeria pomeroyi
Source: Microbiol Spectr. 2022 Oct 27;10(6):e03191-22. doi: 10.1128/spectrum.03191-22 (PMC9769926; doi:10.1128/spectrum.03191-22)

Oxidative stress regulates a pivotal metabolic switch in dimethylsulfoniopropionate degradation by the marine bacterium *Ruegeria pomeroyi*

Tao Wang, Qiuyuan Huang, Andrew S Burns, Mary Ann Moran, William B. Whitman

Supplementary Materials

Quantification of H_2O_2

For measuring H_2O_2 in reservoir, the absorbance at 240 nm of the H_2O_2 solution was measured with a 1.16 mM KH_2PO_4 solution as the control. The concentration of H_2O_2 was calculated from the molar extinction coefficient of 43.6 $M^{-1}cm^{-1}$ at 240 nm.

For measuring H_2O_2 in the chemostat outflow, the FOX1 reaction was used with sorbitol to increase sensitivity (Wolff 1994). Chemostat outflow was collected on ice for 15 minutes to minimize the catalase activity during the collection. To completely inactivate catalase and adjust the sample pH, 975 µl of the outflow was acidified with 25 µl 1 M H_2SO_4. Cellular debris were removed by centrifuged at 5,000 g for 5 min. A series of 0-50 µM H_2O_2 standard samples was prepared by diluting fresh 1 mM H_2O_2 in sterile medium acidified as above. 200 µl of FOX1 reagent [100 µM xylenol orange, 250 µM $(NH_4)_2Fe(SO_4)_2$, 100 mM sorbitol in 25 mM H_2SO_4] was mixed with 20 µl of each sample in duplicate for the standard or triplicate for the chemostat outflow. After incubating at room temperature for 20 minutes, the absorbance at 595 nm was read in a plate reader (BioTek Synergy Mx).

Catalase assay

The outflow was collected and assayed immediately. Cell dry weight was calculated as previously described (Koch 2007), and the protein was estimated as 55% of the cell dry weight. To measure the enzyme activity of chemostat culture, the fresh outflow was collected and assayed immediately. Cell dry weight was calculated from the absorbance at 660 nm by the equation: dry weight (μg/ml) = $364.74A_{660} + 6.7A_{660} \times A_{660}$. The protein was estimated as 55% of the cell dry weight. The assays were set as described by Chance and Maehly with modification (Chance and Maehly 1955). Briefly, culture was directly mixed with equal volume of 0.05% (v/v) H_2O_2 in 50 mM potassium phosphate buffer (pH 7.0). The absorbance at 240 nm was monitored for 3 minutes at 30 °C. The H_2O_2 concentration was calculated from the molar extinction coefficient.

Bioinformatic analysis

A total of 9.569 to 14.848 million (M) uniquely mapped clean reads were obtained for the subsequent analyses (Supplementary Table 4). The average Q30 was 94.06%, and the lowest was 93.08%. The average GC content was 64.09 mol %, which was close to the genome GC content of 64.1 mol %. The average overall alignment rate without sample K1 was 98.87 % with a range of 95.21-100 %. Sample K1 had a low overall alignment rate of 82.22 %, and it was found to have a 16.32 % overall alignment rate to the *Escherichia coli* genome (K-12 substr. MG1655). As no contamination was found during growth, this contamination appeared to have occurred during the downstream processing and should not have affected the reliability of the *R. pomeroyi* reads, which were included in the subsequent analyses.

The reads of all 4457 genes were counted by featureCounts and then processed by DEseq2. The PCA (Figure 3) shows that among all the conditions, often one replicate did not cluster with the other replicates. One possible reason leading to these differences could be batch effects during sample processing. For example, some samples failed the quality check for library construction so that additional replicate samples were prepared from different chemostat runs. Similarly, some replicates were sequenced at different times. These 'batch' effects may have contributed to the observed differences between the replicates. To look for batch effects, the distances between replicates were calculated from the regularized-logarithm transformations (rlog) of the raw counts of all 24 samples (Supplementary Table 5). For 14 replicates, there were no batch differences in their processing, and the average difference (± standard deviation) was 35±18. Six replicates differed only in the sequencing run, and the average difference was 42±18. Lastly, four replicates differed in both the chemostat run and RNA purification procedure, and the average difference was 48±10. Because the differences were not significantly different between replicates processed in different batches, the batch effects were likely to be small.

Upon calculation of the differential expressed genes (DEGs) for each of the comparisons in Figure 2, it was found thar the number of DEGs was greatly reduced for comparisons including replicate WD1. When WD1 was dropped in subsequent analyses, the number of DEGs was comparable to other comparisons even though only two replicates were used for the WD condition. When comparing different conditions to the wild-type with no additions (W transcriptome), 23 samples without WD1 were processed by DESeq2 together and each condition was chosen to be the experimental condition with wild-type with no additions as the control condition. A shrinkage estimator was not used.

For PCA, all 23 samples were normalized together first by DESeq2. The results were calculated and plotted by DESeq2 by the normalized counts of selected genes.

Supplementary Table 1. Primers used for generating pCR2.1 deletion vector. Restriction sites are in bold.

Primers	Sequences
*tetAR*_F SpeI	5’-**ACTAGT**ACCGTATTACCGCCTTTGAGTGAG-3’
*tetAR*_R XhoI	5’-**CTCGAG**ACGCTGAGTGCGCTTCAAATCATC-3’
*katG*_Up_Fwd HindIII	5’-GACCATGATTACGCC**AAGCTT**GGACACCAACATGCCCAG-3’
*katG*_Up_Rev SpeI	5’-GGCGGTAATACGGT**ACTAGT**CAGCACCAGAAAGAACCGG-3’
*katG*_Down_Fwd XhoI	5’-AGCGCACTCAGCGT**CTCGAG**GACCTAGGCCGCGCGTCCCG-3’
*katG*_Down_Rev XbaI	5’-GGGCGAATTGGGCCC**TCTAGA**CTGGGCATGTTGGTGTCC-3’

Supplementary Table 2. H_2O_2 stability in culture media[1].. The abiotic chemostat controls followed the same procedure for the H_2O_2 addition except that a starter culture was not added. The carbon sources were 2 mM glucose or 2 mM glucose plus 0.25 mM DMSP. The maximum volume was 144 ml. The flowrates of medium and air were 0.1 and 3 ml min^{-1}, respectively. After two volume changes, H_2O_2 was applied to the phosphate solution. The H_2O_2 and DMSP in the outflow were measured twice daily.

H_2O_2 added	1000	1000	100	100
DMSP added[2]	0	250±16	0	250±16
H_2O_2 in outflow[3]	606±54	704±26	49±3	66±5
DMSP in outflow[4]	0	231±20	0	233±19

[1]All values are reported in micromolar.

[2]The DMSP concentration added was the average of 6 measurements at the beginning and the end of the experiment. The 95% confidence intervals are indicated. Note the DMSP concentrations in reservoir measured before experiment are 525.5, 532.5 and 512.0 µM. After the experiment, the measurements are 516.5, 457.7 and 453.6 µM. By T-test, the p value is 0.0889. The difference is considered to be not statistically significant.

[3]The concentration is the average detected from day 3 to day 5 after adding H_2O_2 (n=9), when the concentration was stable. The 95% confidence intervals are indicated. In the absence of added H_2O_2, the concentration measured was <0.5 µM.

[4]The amount of DMSP in outflow was measured in triplicates at the end of the experiment. The 95% confidence intervals are indicated.

Supplementary Table 3. Catalase specific activity of *R. pomeroyi* wild type strain in chemostat during growth with glucose or glucose plus DMSP with hydrogen peroxide addition after the measurement on day 0.[1].

Days	Glucose	Glucose + DMSP
Day 0	11.5±0.5	6.9±0.2
Day 1	21.5±0.4	19.3±0.4
Day 2	23.8±0.3	21.0±0.1
Day 3	24.6±0.4	23.0±0.5
Day 4	24.3±0.3	23.5±0.2
Day 5	24.1±0.5	23.9±0.1

[1]: all values are reported in nanomole per minute per microgram protein. The error indicates the 95% confidence intervals.

Supplementary Table 4. Summary of sequencing data including the number of reads and indicators of their quality. Sample abbreviations are defined in Figure 2, with the number indicating the replicate.

Sample	Raw reads (M)	Effective rate (%)	Q30 (%)	GC (%)	Overall alignment rate (%)
W1	9.706	98.10	94.27	62.64	98.06
W2	11.170	97.71	94.97	63.55	97.68
W3	11.967	96.85	95.07	63.02	95.21
WH1	9.569	97.19	96.13	63.00	99.63
WH2	9.977	96.76	95.90	63.13	99.62
WH3	9.705	97.28	95.91	62.81	98.64
WD1[1]	10.198	99.25	93.94	66.78	99.57
WD2	14.037	98.33	94.04	63.70	99.00
WD3	12.162	99.11	93.91	63.46	100.00
WDH1	11.159	98.77	93.62	65.39	98.93
WDH2	10.276	99.00	94.35	65.82	99.18
WDH3	12.441	98.95	93.70	63.61	98.28
K1	11.742	97.46	95.18	61.79	82.22
K2	13.889	95.36	95.15	63.72	99.12
K3	14.848	98.57	94.69	64.50	99.13
KH1	11.661	98.48	93.33	64.92	98.84
KH2	12.218	98.68	93.08	65.86	98.95
KH3	12.448	98.45	93.35	63.59	98.88
KD1[2]	10.688	99.07	95.05	64.93	99.40
KD2	13.053	99.20	93.68	64.24	99.07
KD3	11.926	99.31	93.81	63.16	98.60
KDH1	13.657	99.39	93.59	66.05	99.25
KDH2	14.674	99.30	93.36	65.31	99.37
KDH3	13.852	99.60	93.69	66.31	99.50

[1] Reads from this sample were subsequently removed from the analysis.
[2]Data contained contaminating reads from *E.coli*, which were removed during the analysis.

Supplementary Table 5. Comparison of replicates and determination of batch effects. To calculate the distance between samples, the counts were transformed by regularized-logarithm transformation (rlog). Sample abbreviations are defined in Figure 2, with the number indicating the replicate. NA: not applicable.

Sample	Chemostat batch	Sequencing batch	Distance to replicate 1	Distance to replicate 2
W1	A	5	NA	37
W2[1]	A[2]	5	37	NA
W3[1]	A[2]	5	43	24
WH1[1]	A	4	NA	12
WH2[1]	A	4	12	NA
WH3[1]	A	4	49	47
WD1	B	3	NA	65
WD2	B	3	65	NA
WD3	B	2	71	14
WDH1	B	3	NA	60
WDH2	B	3	60	NA
WDH3	B	2	37	47
K1[1]	C[2]	5	NA	15
K2[1]	C[2]	5	15	NA
K3	C	5	56	56
KH1	C	3	NA	43
KH2	C	3	43	NA
KH3	C	3	24	48
KD1	D	1	NA	39
KD2	D	2	39	NA
KD3	D	2	45	17
KDH1	D	2	NA	21
KDH2	D	2	21	NA
KDH3	D	2	11	28

[1] After purification by the normal method, samples were further purified with the RNA Clean & Concentrator kit. [2] Samples prepared from a different chemostat run.

Supplementary Table 6. Number of differentially expressed genes in each comparison with adjusted p values <0.1. Abbreviations for growth conditions are defined in Figure 2. Conditions in parentheses are controls for each comparison. The mean count for all genes under all conditions was 1 or greater.

	K(W)	WH(W)	KH(K)	WD(W)	KD(K)	KD(WD)	WDH (WD)	KDH (KD)	WDH (WH)	KDH (KH)
Upregulated	73	65	360	634	775	668	869	543	1084	1551
Downregulated	20	97	422	793	862	248	325	1417	1055	1514
Outliers[1]	52	24	26	8	19	0	0	0	9	0

[1]Detected by Cook's distance.

Supplementary Table 7. Full list of genes selected for analyzing sulfur metabolism and oxidative stress.

Locus tag	Genes	Annotations
		Oxidative stress genes (84)
SPO_RS10675		hydrogen peroxide-inducible genes activator (*oxyR* homologue)
SPO_RS11345		hydrogen peroxide-inducible genes activator (*oxyR* homologue)
SPO_RS20080	*katG*	catalase/peroxidase HPI
SPO_RS00535	*yaaA*	peroxide stress protein YaaA
SPO_RS19660	*trxA*	thioredoxin
SPO_RS17330	*trxA*	thioredoxin
SPO_RS04550	*trxB*	thioredoxin-disulfide reductase
SPO_RS13145		thioredoxin domain-containing protein
SPO_RS04995	*soxW*	thioredoxin family protein
SPO_RS02235		thiol reductase thioredoxin
SPO_RS10235		SufD family Fe-S cluster assembly protein
SPO_RS10240	*sufC*	Fe-S cluster assembly ATPase SufC
SPO_RS10265	*sufB*	Fe-S cluster assembly protein SufB
SPO_RS13285		SUF system Fe-S cluster assembly protein
SPO_RS13290		iron-sulfur cluster assembly accessory protein
SPO_RS06770	gor	glutathione-disulfide reductase
SPO_RS03235		carboxymuconolactone decarboxylase family protein/alkylhydroperoxidase AhpD family core domain protein
SPO_RS03835		carboxymuconolactone decarboxylase family protein/alkylhydroperoxidase AhpD family protein
SPO_RS06395		peroxidase-related enzyme
SPO_RS07105		carboxymuconolactone decarboxylase family protein/alkylhydroperoxidase AhpD family protein
SPO_RS19070		peroxidase-related enzyme
SPO_RS19230		carboxymuconolactone decarboxylase family protein/alkylhydroperoxidase AhpD family protein
SPO_RS00370	*hemH*	protoporphyrin/coproporphyrin ferrochelatase
SPO_RS01680	*ccpA*	cytochrome-c peroxidase
SPO_RS03770		peroxidase

SPO_RS04325		cytochrome-c peroxidase
SPO_RS18990		glutathione peroxidase
SPO_RS20600		di-heme cytochrome c peroxidase family protein/hypothetical protein
SPO_RS21190		methylamine utilization protein MauG/c-type cytochrome
SPO_RS22125		vanadium-dependent haloperoxidase
SPO_RS20725		LysR family transcriptional regulator
SPO_RS04195		LysR family transcriptional regulator
SPO_RS17675		LysR family transcriptional regulator
SPO_RS07845		LysR family transcriptional regulator
SPO_RS16785		hydrogen peroxide-inducible genes activator
SPO_RS21470		LysR family transcriptional regulator
SPO_RS21795		LysR family transcriptional regulator
SPO_RS20665		LysR family transcriptional regulator
SPO_RS01230		LysR family transcriptional regulator
SPO_RS20010	*pcaQ*	pca operon transcription factor PcaQ
SPO_RS12080		LysR family transcriptional regulator
SPO_RS10920	*lexA*	transcriptional repressor LexA,in the response to DNA damage (SOS response)
SPO_RS18960		peroxiredoxin
SPO_RS01605	*soxR*	redox-sensitive transcriptional activator SoxR
SPO_RS04980	*soxR*	winged helix-turn-helix transcriptional regulator
SPO_RS04985	*soxS*	regulatory protein
SPO_RS11860	*sodB*	superoxide dismutase
SPO_RS00070	*msrA*	peptide-methionine (S)-S-oxide reductase MsrA
SPO_RS18980	*msrA*	peptide-methionine (S)-S-oxide reductase MsrA
SPO_RS18985	*msrB*	peptide-methionine (R)-S-oxide reductase MsrB
SPO_RS16560	*msrQ*	protein-methionine-sulfoxide reductase heme-binding subunit MsrQ
SPO_RS16565	*msrP*	protein-methionine-sulfoxide reductase catalytic subunit MsrP
SPO_RS10320	*recA*	recombinase RecA
SPO_RS00775	*recF*	DNA replication/repair protein RecF

SPO_RS08535	*recG*	ATP-dependent DNA helicase RecG
SPO_RS08815	*recJ*	single-stranded-DNA-specific exonuclease RecJ
SPO_RS16190	*recO*	DNA repair protein RecO
SPO_RS18080	*recR*	recombination protein RecR
SPO_RS15785	*ruvB*	Holliday junction branch migration DNA helicase RuvB
SPO_RS15790	*ruvA*	Holliday junction branch migration protein RuvA
SPO_RS15795	*ruvC*	crossover junction endodeoxyribonuclease RuvC
SPO_RS15845	*priA*	primosomal protein N'
SPO_RS19505	*polA*	DNA polymerase I
SPO_RS11250	*uvrA*	excinuclease ABC subunit UvrA
SPO_RS02750	*uvrB*	excinuclease ABC subunit UvrB
SPO_RS18425	*uvrC*	excinuclease ABC subunit UvrC
SPO_RS05950	*uvrD*	UvrD-helicase domain-containing protein
SPO_RS08530	*ligA*	NAD-dependent DNA ligase LigA
SPO_RS10520	mfd	transcription-repair coupling factor
SPO_RS07205		DNA modification methyltransferase
SPO_RS00750	*mutM*	bifunctional DNA-formamidopyrimidine glycosylase/DNA-(apurinic or apyrimidinic site) lyase
SPO_RS17455	*mutY*	A/G-specific adenine glycosylase
SPO_RS00305	*mutT*	8-oxo-dGTP diphosphatase MutT
SPO_RS00055	*mutS*	DNA mismatch repair protein MutS
SPO_RS17030	*mutL*	DNA mismatch repair endonuclease MutL
SPO_RS18140	nth	endonuclease III
SPO_RS12740	xth	exodeoxyribonuclease III
SPO_RS09650	tag	DNA-3-methyladenine glycosylase I
SPO_RS01465	*udgA*	uracil-DNA glycosylase
SPO_RS10785		DNA-3-methyladenine glycosylase 2 family protein
SPO_RS06850	xseA	exodeoxyribonuclease VII large subunit
SPO_RS01270	xseB	exodeoxyribonuclease VII small subunit
SPO_RS10390	zwf	glucose-6-phosphate dehydrogenase

SPO_RS15375	zwf	glucose-6-phosphate dehydrogenase
		Sulfur metabolism genes (39)
SPO_RS00195	*cysQ*	3'(2'),5'-bisphosphate nucleotidase CysQ
SPO_RS11395	*cysK*	pyridoxal-phosphate dependent cysteine synthase A
SPO_RS11400	*cysE*	serine O-acetyltransferase
SPO_RS13360	*cysJI*	nitrite/sulfite reductase
SPO_RS13365	*cysH*	phosphoadenylyl-sulfate reductase
SPO_RS04535	sat	bifunctional sulfate adenylyltransferase/adenylylsulfate kinase
SPO_RS04990	*soxV*	cytochrome C biogenesis protein CcdA
SPO_RS04995	*soxW*	thioredoxin family protein
SPO_RS05000	*soxX*	sulfur oxidation c-type cytochrome SoxX
SPO_RS05005	*soxY*	thiosulfate oxidation carrier protein SoxY
SPO_RS05010	*soxZ*	thiosulfate oxidation carrier complex protein SoxZ
SPO_RS05015	*soxA*	sulfur oxidation c-type cytochrome SoxA
SPO_RS05020	*soxB*	thiosulfohydrolase SoxB
SPO_RS05025	*soxC*	sulfite dehydrogenase, sulfur oxidation molybdopterin C protein
SPO_RS05030	*soxD*	c-type cytochrome
SPO_RS05035	*soxE*	c-type cytochrome
SPO_RS05040	*soxF*	FAD-dependent oxidoreductase
SPO_RS07755	*soxY*	quinoprotein dehydrogenase-associated SoxYZ-like carrier
SPO_RS14335	*soxH*	MBL fold metallo-hydrolase
SPO_RS17005	*soeC*	LysR family transcriptional regulator/membrane-bound sulfite dehydrogenase soeABC
SPO_RS17010	*soeB*	serine/threonine protein phosphatase/membrane-bound sulfite dehydrogenase soeABC
SPO_RS17015	*soeA*	L-threonine 3-dehydrogenase/membrane-bound sulfite dehydrogenase soeABC
SPO_RS18875	*sseA*	3-mercaptopyruvate sulfurtransferase
SPO_RS03405	*tauA*	ABC transporter substrate-binding protein/taurine transporter
SPO_RS03410	*tauB*	ABC transporter ATP-binding protein/taurine transporter
SPO_RS03415	*tauC*	ABC transporter permease subunit/taurine transporter
SPO_RS09100		sulfate/tungstate uptake family ABC transporter, permease protein

SPO_RS09105		ATP-binding cassette domain-containing protein/sulfate/tungstate uptake family ABC transporter
SPO_RS09110		extracellular solute-binding protein/sulfate/tungstate uptake family ABC transporter
SPO_RS09930	*sulP*	sulfate permease
SPO_RS15495	*sulP*	SulP family inorganic anion transporter
SPO_RS09750		cysteine synthase A
SPO_RS08200		NAD(P)/FAD-dependent oxidoreductase/sulfide:quinone oxidoreductase
SPO_RS08800	*metA*	homoserine O-succinyltransferase
SPO_RS06880	*metZ*	O-succinylhomoserine sulfhydrylase
SPO_RS21430	*megL*	methionine gamma-lyase
SPO_RS08355		selenium-binding protein
SPO_RS07295	*metY*	O-acetylhomoserine aminocarboxypropyltransferase/cysteine synthase
SPO_RS09575	MTR	5-methyltetrahydrofolate-homocysteine methyltransferase

Supplementary Figure 1. Chemostat design for H_2O_2 additions. One of the reservoirs contained 2X medium of 160 mM HEPES (pH 6.8), 0.01 mM Fe(III)EDTA, 0.2 % (v/v) trace mineral solution, and 0.2 % (v/v) vitamin solution in general salts solution with carbon sources. The other reservoir contained 1.16 mM KH_2PO_4 solution with the desired H_2O_2 concentration. The two reservoirs were connected by a single pump, and the flowrate was 0.05 ml/min for a combined flow rate at the chemostat of 0.1 ml/min and leading to the final Fe(III)EDTA concentration of 5 μM. Air was bubbled into the chemostat at 3 ml/min. The stir bars in reservoirs and the culture are not shown.

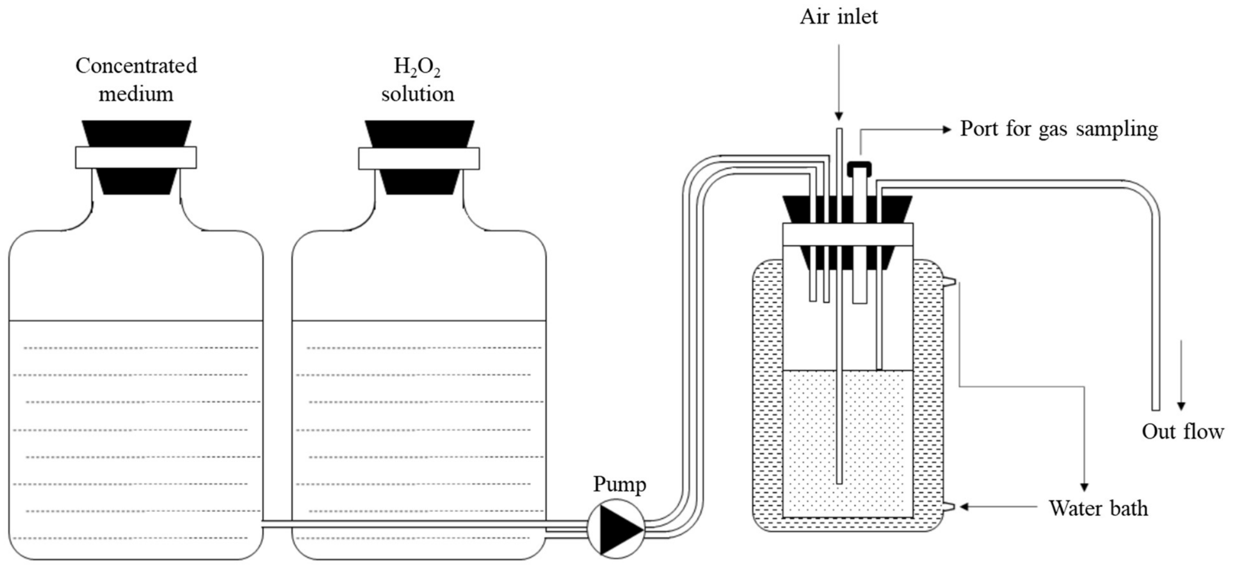

Supplementary Figure 2. Cell density chemostat with increasing H_2O_2 concentrations. These chemostat were performed with a seawater-based minimal medium containing 68 µM Fe(III)EDTA (Wirth et al. 2020). The sole carbon source was 2 mM glucose. The concentration of H_2O_2 addition was labeled at the beginning of each stage, which was indicated by the black lines. A. optical density of the wild-type culture; B. optical density of the culture of the *ΔkatG* mutant.

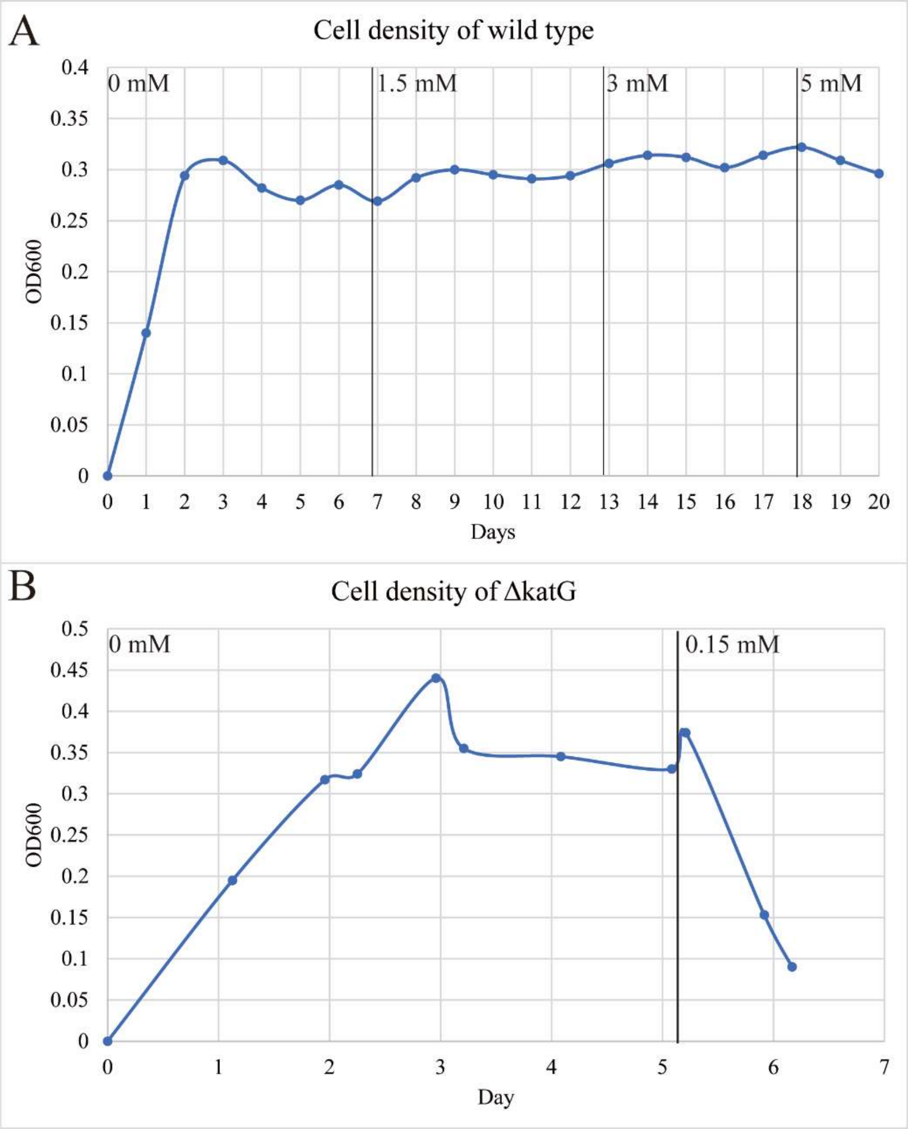

Supplementary Figure 3. Identification of oxidative stress responsive genes. Heatmap of genes likely to play a role in the oxidative stress response in *R. pomeroyi* based upon their annotation and homology to oxidative stress responsive genes in other proteobacteria. For the four homologs of *E.coli oxyR*, the abundance of transcripts from one of them (SPO_RS20725) was very low and considered an unreliable indicator for that reason. The abundance of transcripts of two others, SPO_RS11345 and SPO_RS16785, did not respond to the addition of H_2O_2 in either the wild-type or the Δ*katG* mutant in the absence of DMSP and were not considered further. Abbreviations are defined in Figure 2.

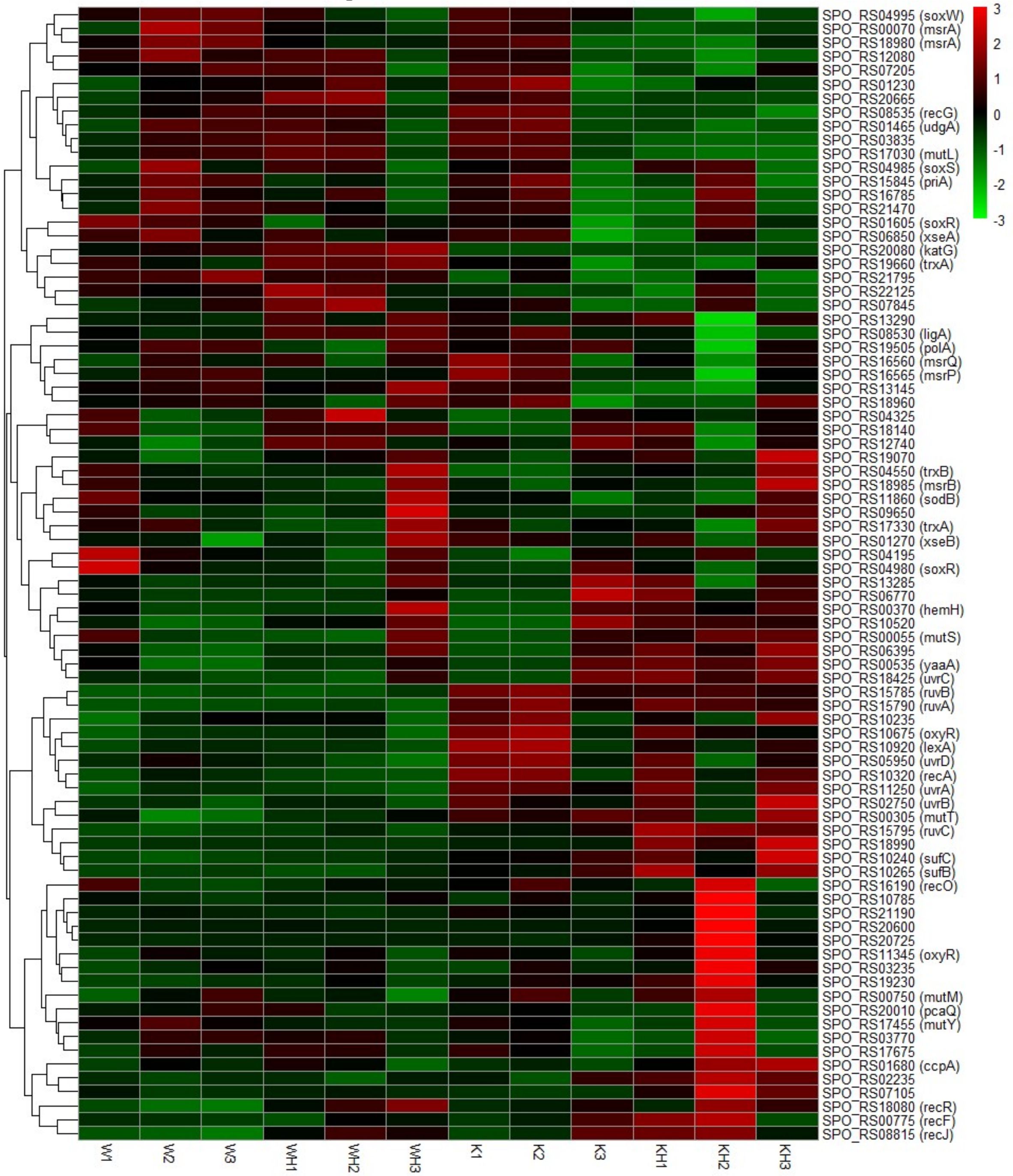

Supplement: Supplemental file 1 — Supplemental material. Download spectrum.03191-22-s0001.pdf, PDF file, 1.4 MB [file spectrum.03191-22-s0001.pdf]
